# Stringent response-mediated ferroptosis-like death resistance underlies *Novosphingobium* persistence during ciprofloxacin stress

**DOI:** 10.1128/aem.01475-25

**Published:** 2025-09-15

**Authors:** Qian Xu, Yili Huang

**Affiliations:** 1 State Key Laboratory of Soil Pollution Control and Safety, Department of Environmental Science, College of Environmental and Resource Sciences, Zhejiang University 601928 , Hangzhou, China; University of Delaware, Lewes, Delaware, USA

**Keywords:** stringent response, antibiotic resistance, *Novosphingobium*, siderophore, oxidative stress, ferroptosis-like death

## Abstract

Antibiotics, as emerging hazardous materials in the environment, pose significant risks to ecosystems and contribute to the spread of antibiotic-resistant bacteria. Although extensive knowledge has been accumulated on antibiotic-resistance mechanisms in individual bacteria, less is understood about how the bacterial communities respond to antibiotic exposure under natural environmental conditions, where nutrient supplies are often limited and fluctuating. Here, we report that *Novosphingobium* dominated in a wetland bacterial community under 1 µg/mL ciprofloxacin (CIP) exposure and persisted during DL-serine hydroxamate-induced starvation, where the stringent response alarmer (p)ppGpp was detected. Metagenome sequencing revealed that genes associated with siderophore transport, cytochrome *c*, and glutathione S-transferase were significantly enriched in *Novosphingobium*, linking its dominance under CIP stress to iron homeostasis and oxidative stress responses. Further study on the survival mechanism of *Novosphingobium pentaromativorans* US6-1 under 8 µg/mL CIP stress demonstrated that stringent response regulated the growth rate and maintained cell viability by suppressing the TCA cycle and oxidative phosphorylation, deterring the entry of CIP and siderophore into cells, reducing intracellular ferrous iron and malondialdehyde, and balancing cellular redox status, thereby protecting cells from ferroptosis-like death. This study is the first to report *Novosphingobium’s* dominance and persistence in a bacterial community during CIP stress in natural environmental conditions and to propose the stringent response-mediated ferroptosis-like death resistance as one of its key survival mechanisms.

**IMPORTANCE:**

Antibiotics in the environment are increasingly recognized as a new class of pollutants that accelerate the evolutionary selection of antibiotic-resistant bacteria. However, little is known about how this selection occurs under natural conditions, including how specific bacteria taxa and mechanisms respond to particular antibiotics. This study reveals for the first time the selection effect of CIP on *Novosphingobium* under nutrient-limited conditions, during which stringent response and iron homeostasis play important roles. An innovative linkage between stringent response and ferroptosis-like death resistance is proposed in *N. pentaromativorans* US6-1, which serves as the CIP resistance mechanism for *Novosphingobium*. These findings may help inform strategies to combat antimicrobial resistance in the natural environment.

## INTRODUCTION

Antibiotic contamination has emerged as a critical global environmental challenge, from which many escalating ecological and public health implications arise. Residual antibiotics persist in 65% of sampled rivers worldwide (1). The fluoroquinolone antibiotic ciprofloxacin (CIP) is one of the most frequently detected due to its extensive consumption, with its highest measured concentration reaching 13,567 ng/L in Buyukcekmece lake, Turkey (2). Besides posing potential ecological risks to aquatic organisms, antibiotics have also accelerated the propagation of antibiotic resistance genes (ARGs) within bacteria themselves. Environmental exposure to antibiotics could therefore lead to selection for antimicrobial resistance (AMR) in microorganisms, and thus contribute to the global AMR crisis (3).

In most natural econiches, microbes inevitably encounter a fluctuation of nutrient supply and antibiotic pollution; it remains unclear whether and how antibiotic exposure interferes with the bacterial community’s response to the nutrient fluctuation and what the mechanisms are for bacteria to survive under this scenario.

The stringent response is a global regulatory system, allowing bacteria to survive in response to nutrient limitations (e.g., amino acid starvation) and other environmental stresses such as antibiotic exposure. The stringent response is mediated by the small-molecule guanosine tetra- or penta-phosphate (p)ppGpp, which is synthesized by the RSH protein family (RelA/SpoT) (4). (p)ppGpp serves as an alarm for stressful situations. As it does so, it binds to transcriptional regulators to inhibit various core pathways of cellular metabolism, including those of DNA and RNA synthesis, ribosome synthesis, and protein production (5).

Previous studies have demonstrated stringent response-mediated antibiotic resistance in various bacteria, including where (p)ppGpp can facilitate DNA break repair by binding to RNA polymerase during ciprofloxacin (CIP) stress (6), where stringent response-regulated cellular tolerance to antibiotics may occur through reduced reactive oxygen species (ROS) production (7, 8); and where stringent response plays a crucial role in biofilm formation, thereby enhancing multidrug resistance (9).

However, while most such studies have been conducted under laboratory conditions using *Escherichia coli* or pathogenic bacteria, in the natural environment, the continuous accumulation of antibiotics in various environmental biomes has potentially enabled a multitude of microbes to become important hosts of resistance genes, their survival likely contributing to the AMR crisis. Furthermore, unique nutrient limitations present in numerous natural environments may render the stringent response a critical mechanism for survival under antibiotic-induced stress. It is therefore highly necessary to explore whether and how stringent response regulates antibiotic resistance in naturally occurring environmental microbes.

In this study, by exposing the bacterial community from Xixi wetland in China to sub-MIC CIP and DL-serine hydroxamate (SHX, used to mimic starvation-induced stringent response), *Novosphingobium* was found to dominate and persist under CIP stress. Results of metagenome analysis suggested that factors of iron homeostasis and oxidative stress occurred as key contributors to this dominance and persistence of *Novosphingobium*. Further investigation on *Novosphingobium pentaromativorans* US6-1 revealed that the stringent response mediated its ferroptosis-like death resistance under CIP stress, thus serving as one of the mechanisms facilitating the persistence of *Novosphingobium* in this natural aquatic system.

## MATERIALS AND METHODS

### Field sample collection and processing

The sampling site was located in Xixi wetland of Hangzhou (30°14′56.3″N, 120°12′18.8″E). Sampling work was conducted in January 2024. Five liters of the water sample was collected from the surface of the wetland, stored in sterile sample bags, and then transported to the laboratory for immediate processing.

Water samples were filtered through a 5 µm membrane, with the filtrate collected for secondary filtering through a 0.22 µm membrane. The 0.22 µm membrane was then rinsed with 1 mL PBS buffer 50 times. PBS buffer was then added to 50 mL R2A liquid medium (Sangon Biotech, Shanghai, China) and incubated at 200 rpm, 30°C until OD_600_ = 1, for preparation of a seed culture.

### Bacterial community treatment

The original bacterial community isolated from the Xixi wetland was designated as WL0, and the bacterial community cultured in the R2A medium was designated as WL1. DL-Serine (500 µM) hydroxamate (SHX) (Sigma-Aldrich, Shanghai, China) was added to the culture during the rapid growth phase to induce metabolic synthesis of (p)ppGpp by amino acid starvation (10). CIP (1 µg /mL) was added to the culture medium representing sub-MIC antibiotic exposure that still permitted continued strain growth (11). The seed culture was sub-cultured into an R2A liquid medium at a dilution of 0.5% and cultivated in a shaker incubator (Zhichu, Shanghai, China).

### Measurements of (p)ppGpp

At the different time points of incubation, cells were separately collected by centrifugation and suspended in 1 mL of 0.9% saline. One hundred microliters of 11 M formic acid was then added, vigorously mixed, and incubated on ice for 30 min with samples centrifuged at 10,000 × *g* at 4°C for 10 min. The supernatant was filtered through a 0.22 µm membrane and stored at −20°C for high-performance liquid chromatography (HPLC) analysis.

HPLC analysis was performed on a high-performance liquid chromatography (LC-20A HPLC system, Shimadzu, Japan) by using a ZORBAX SB-C18 column (4.6 × 250 mm, 5 µm, Agilent) at 40°C. The flow phase (pH = 6) consisted of 125 mM KH2PO4, 10 mM tetrabutylammonium dihydrogen phosphate, 60 mL/L methanol, and 1 g/L KOH. The flow rate was 1.0 mL/min. (p)ppGpp was monitored using a UV detector at 254 nm and identified by comparison to a retention time of 100 µM (p)ppGpp standards (TriLink Biosciences, San Diego, USA).

### 16S rRNA gene amplicon sequencing and data analysis

Cells with or without SHX treatment were cultured in the R2A liquid medium under shaking conditions and harvested at 50 h. CIP (1 µg/mL) was then added to the culture according to experimental designs. The bacterial cells were centrifuged at 10,000 × *g* at 4°C for 10 min, and after treatment with liquid nitrogen for 15 min, cells were then stored at −80°C. 16S rRNA gene amplicon sequencing was performed at Novogene Co., Ltd.

The 16S rRNA genes of the V3–V4 hypervariable region were amplified using forward primer 341F (5′-CCTAYGGGRBGCASCAG-3′) and reverse primer 806R (5′-GGACTACNNGGGTATCTAAT-3′). The PCR amplicons were pooled and sequenced using an Illumina NovaSeq 6000 platform. The raw sequencing data were processed using the fastp software (Version 0.23.1) and compared with the species annotation database (Silva database https://www.arb-silva.de/ for 16S). QIIME2 (Version QIIME2-202202) was used for denoising and species annotation of clean data. The sequenced 16S rRNA gene clean data were submitted to the NCBI with the accession number PRJNA1198712. Finally, the data of each sample were normalized; alpha diversity analysis and beta diversity analysis were based on the normalized data. Statistical analysis methods, including *t*-test, MetagenomeSeq, and linear discriminant analysis effect size (LEfSe), were used to demonstrate significant differences among the groups of samples.

### Metagenomic sequencing and data analysis

Cells were harvested at 50 h. Metagenomic sequencing was performed at Novogene Co., Ltd. (Beijing, China).

After DNA extraction, the genomic DNA samples were fragmented into short fragments, then end-polished, A-tailed, and ligated with full-length adapters for Illumina sequencing. After purification and PCR amplification, the resulting library was initially quantified on the Qubit 2.0 (Thermo Fisher Scientific) and assessed using an Agilent Fragment Analyzer System (Agilent). The qualified libraries were pooled and sequenced using the Illumina platform PE150.

The FASTP software was used to preprocess the raw data to obtain clean data for subsequent analysis. The filtered sequences were aligned using the software Bowtie2. The MEGAHIT software was used for assembly analysis of clean data. Open reading frame (ORF) prediction was performed using the MetaGeneMark CD-HIT software to eliminate the redundancy of the ORF prediction results. The ORF prediction results were then realigned using the software Bowtie2 to obtain the final gene catalog.

The gene catalogs were then compared with the Micro_NR database (https://www.ncbi.nlm.nih.gov/) to obtain the species annotation information of each gene (unigene). The species abundance table at different taxonomic levels was obtained by combining the gene abundance table.

The DIAMOND software (https://github.com/bbuchfink/diamond/) was used to align unigenes with those in the functional database. Functional databases include the KEGG database (http://www.kegg.jp/kegg/) and eggNOG database (http://eggnogdb.embl.de/app/home).

Based on the abundance table at each taxonomic level, statistics of the annotated genes, overview of the relative abundance, and clustering heat map of abundance were carried out. Metabolic pathway comparative analysis, MetagenomeSeq analysis, and linear discriminant analysis effect size method were performed to explore the differences in species and functional compositions among the samples.

### Bacterial strains and culture conditions

The bacterial strains used in this work were *Novosphingobium pentaromativorans* US6-1 wild-type strain (WT), the stringent response gene *rsh* deletion mutant (Δ*rsh*), and its complementation strain (P*rsh*). All strains were sourced from collections within our own laboratory (12).

1% P5Y3-MM2 medium (0.05 g/L bacteriological peptone, with 0.03 g/ L yeast extract, 25 g/L sea salt, and 1 µM FeSO_4_·7H_2_O, 100 µM KH_2_PO_4_, and 100 µM Na_2_HPO_4_) was used as the poor medium. In accordance with our experimental designs, CIP was added to the culture medium when required. A bacterial culture without antibiotics was set as the control.

A single colony of strains was inoculated in P5Y3 medium and incubated at 200 rpm, 30°C until OD_600_ = 1 to prepare as seed culture. Cells were then collected by centrifugation (1,698 g, 10 min) and rinsed three times with 0.9% saline before subculturing to the poor medium at a dilution of 0.5%.

### Measurements of the diameter of inhibition zone and minimal inhibitory concentration (MIC)

An antimicrobial susceptibility testing (AST) was performed according to the United States Clinical and Laboratory Standards Institute (CLSI) method as the pilot experiment. Each single colony strain was inoculated in P5Y3 medium and incubated at 200 rpm, 30°C until OD_600_ = 0.2 (1 × 10^8^ CFU/ml). The cells were collected by centrifugation (1,698 g, 10 min), washed with 0.9% saline three times, with 100 µL of the bacterial solutions then plated on the 1% P5Y3 (poor) or P5Y3 (rich) medium agar plates. The antibiotic disks (Bkmam, Changde, China) were placed in the middle of the plate in triplicates. All the agar plates were cultured at 30°C for 3 days. The diameters of the inhibition zones were then manually measured by a ruler and plotted using the software GraphPad Prism 10.

The seed culture of the US6-1 WT was sub-cultivated at a 1% dilution into the 1% P5Y3 medium. The CIP concentrations were set as 512, 256, 128, 64, 32, 16, 8, 4, 2, and 1 µg/mL by serial dilution. The tubes with bacterial inoculation but without antibiotics served as the positive control, while the tubes with sterile culture medium only served as the negative control. The tubes were shaken at 200 rpm while being incubated at 30°C. The growth of bacteria was judged visually by the turbidity of the broth. Results were valid only when the positive control tube was turbid and the negative control tube was clear.

### Determination of growth curve and colony-forming unit (CFU) at a sub-MIC antibiotic level

Every culture was cultivated in a shaker incubator, and the OD_600_ was measured at regular time intervals. The growth curves were plotted using the software GraphPad Prism 10. CFU counting was conducted to evaluate the cell viability. One milliliter of the bacterial solution was taken every 24 h, serially diluted with 0.9% saline, and plated on appropriate agar plates. The colony number was counted after incubation at 30°C for 3 days.

### Measurements of extracellular ciprofloxacin contents

The bacterial culture was centrifuged at 1,698 × *g* for 10 min to obtain the supernatant, and 0.5 mL each of the supernatant was mixed with 1 mL acetonitrile (ACN). The supernatant was then filtered through a 0.22 µm membrane and stored at 4°C for HPLC analysis.

A stock solution at a concentration of 100 µg/mL for CIP was prepared and stored at 4°C. All working standards were freshly prepared by appropriate dilutions of the stock solution and mixed with ACN (1:2 vol/vol) to plot the standard curve.

HPLC analysis was performed on a high-performance liquid chromatography (LC-20A HPLC system, Shimadzu, Japan) using a TC-C18 column (4.6 × 250 mm, 5 µm, Agilent). The flow phase A was 0.1% trifluoroacetic acid (TFA), and the flow phase B was ACN. The processes of gradient elution are listed in Table S1. The flow rate was 1.0 mL/min. The injected sample volume was 100 µL. The column effluent was monitored at 275 nm for CIP.

### Transcriptome sequencing

For transcriptome sequencing, the WT and Δ*rsh* strains were cultured in the 1% P5Y3 medium with sub-MIC CIP under continuous shaking and harvested at the end of the exponential phase. Bacterial cells were collected and centrifuged at low speed to remove the culture medium and rinsed three times with PBS buffer. The bacteria were collected in 2 mL centrifuge tubes, treated with liquid nitrogen for 15 min, and then stored at −80°C. Transcriptome sequencing was performed at Novogene Co., Ltd. Clean data were obtained after data quality control from raw data. All the downstream analyses were based on the clean data of high quality. Differentially expressed gene (DEG) analysis was performed using DESeq2 (13). Genes with an adjusted *P*-value of less than 0.05, as found by DESeq2, were assigned as differentially expressed. The KOBAS software was used to test the statistical enrichment of DEGs in KEGG pathways. KEGG pathways with corrected *P*-values less than 0.05 were considered significantly enriched by DEGs.

To plot the heatmap of gene expression data, the H-cluster method was used for clustering: taking log_2_ (fpkm +1) for the expression levels of DEGs, and then zero-centered correction was used. The differential genes were divided into several clusters, and the genes in the same cluster had similar trends under different treatment conditions.

### Measurements of siderophore units

The culture was centrifuged at 10,000 × *g* for 10 min to obtain the supernatant, and 0.5 mL of each supernatant was separately mixed with 0.5 mL Chrome Azurol S (CAS) dye solution (14), using uninoculated liquid medium as a reference. The sample (As) and reference (Ar) absorbances at 630 nm were measured by using a spectrophotometer after 1 h incubation at room temperature. Siderophore units are defined as [(Ar-As)/Ar] × 100%.

### Determination of siderophore type

The type of siderophore was determined using standard methods. Hydroxamates were determined using an FeCl_3_ experiment. Catecholates were determined using the Arnow experiment. Carboxylates were determined using the spectrophotometric test (15).

### Measurements of intracellular ferrous iron levels

Bacterial cells were harvested at the end of the exponential phase. Ferrous iron levels were detected by using the ferrous iron content detection kit (Solarbio, Beijing, China) according to the manufacturer’s instructions. Briefly, ferrous iron forms a blue complex with tripyridyltriazine under acidic conditions, with an absorption peak at 593 nm. The content of Fe(II) can be calculated by measuring the absorbance at this wavelength.

### Determination of the oxidative stress response

The oxidative stress response was assessed by measuring reactive oxygen species (ROS) such as O_2_^−^, ·OH, H_2_O_2_, etc., reduced glutathione (GSH), and nicotinamide adenine dinucleotide (NAD). Bacterial cells were harvested at the end of the exponential phase. ROS, GSH, and NAD(H) were measured by using a Reactive Oxygen Species Assay Kit (Beyotime, Shanghai, China), reduced Glutathione (GSH) Content Assay Kit, and NAD(H) Content Assay Kit (Sangong Biotech, Shanghai, China) according to the manufacturer’s instructions.

### Measurements of malondialdehyde (MDA)

WT, Δ*rsh,* and P*rsh* were cultured in the 1% P5Y3 medium with sub-MIC CIP and harvested at the end of the exponential phase under shaking conditions. The cells were broken by sonification (200 W power, sonification for 3 s with an interval of 10 s, repeated 30 times) after adding the extraction solution. The supernatant was then centrifuged (8,000 × *g*, 4°C) for 10 min and placed on ice to be tested. MDA was measured using an MDA Content Assay Kit (Sangong Biotech, Shanghai, China).

### Statistical analyses

All experiments included three biological replicates. Tukey Honestly Significant Difference (HSD) was applied to assess significant differences among different treatments by SPSS Statistics Version 27.0 (IBM Corp.). A *P*-value less than 0.05 was considered statistically significant.

## RESULTS AND DISCUSSION

### Dominance and persistence of *Novosphingobium* under ciprofloxacin stress in the simulated aquatic system

**Fig 1:**
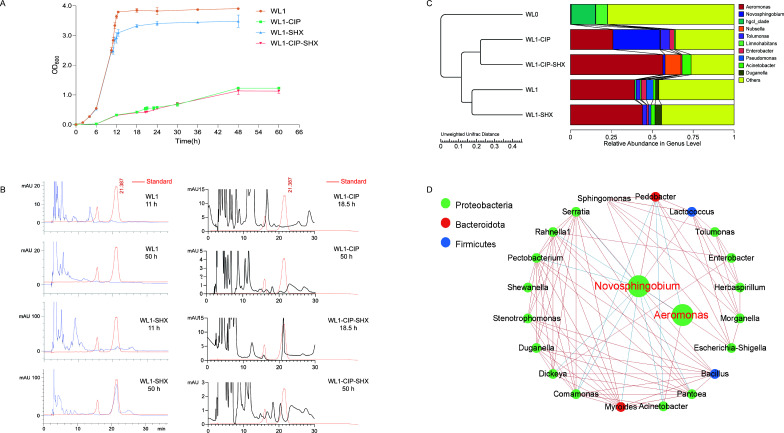
Dominance and persistence of *Novosphingobium* under ciprofloxacin (CIP) stress in a laboratory-based mimic of a natural water system. (**A**) Growth curves of water microbiota from Xixi wetland with or without DL-serine hydroxamate (SHX) or/and CIP treatments in an R2A medium. SHX was added to bacterial culture WL1 and WL1-CIP at 10.5 h and 18 h respectively, to mimic a starvation-induced stringent response. (**B**) Measurement of (p)ppGpp production by HPLC in different bacterial communities at different time points of incubation. (**C**) Cluster analysis for bacterial community compositions at the genus level; (**D**) Community network analysis of bacterial communities from Xixi wetland.

The growth curves of the bacterial communities with different treatments are presented in Fig. 1A‌‌. The bacterial community WL1 showed a typical growth curve in which after a few hours’ lag, a rapid increase in biomass was observed, followed by a plateau. Supplementation with 500 µM SHX to WL1 (designated as WL1-SHX) at 10.5 h induced significant growth inhibition, resulting in lower biomass of WL1-SHX than that of WL1. When WL1 was exposed to CIP (designated as WL1-CIP), the bacterial growth was dramatically inhibited, resulting in much lower biomass. The addition of SHX (designated as WL1-CIP-SHX) at 18 h further inhibited the growth. The biomass of both WL1-CIP and WL1-CIP-SHX increased slowly and steadily in 48 h and then arrived at a plateau.

Measurement of (p)ppGpp indicated that (p)ppGpp was not detected in WL1 or WL1-CIP at different time points of incubation. However, 30 min after the addition of SHX, (p)ppGpp production was detected in both WL1-SHX and WL1-CIP-SHX samples, with the characteristic (p)ppGpp peak emerging at approximately 21 min (Fig. 1B). After 50 h of incubation, (p)ppGpp remained detectable in both WL1-SHX and WL1-CIP-SHX samples. These results indicated that SHX can successfully induce a stringent response in bacterial communities by promoting (p)ppGpp production.

16S rRNA gene amplicon sequencing analysis indicated that the bacterial community structure was sensitive to R2A enrichment and CIP treatment. In this, WL0 was out-grouped from the others (Fig. 1C). After culture in R2A liquid medium, the most abundant bacterial genus was *Aeromonas* (39.23%) in WL1. The addition of SHX did not dramatically change the community structure since WL1-SHX clustered with WL1. However, the addition of sub-MIC CIP significantly changed the community structure of WL1, where *Novosphingobium* (28.71%) became the most abundant genus in WL1-CIP, followed by *Aeromonas* (25.96%). The addition of SHX to WL1-CIP further altered the composition of the bacterial community. Despite this, some *Novosphingobium* (< 2%) still persisted.

Community network analysis indicated that the top two abundant genera, *Novosphingobium* and *Aeromonas,* were negatively correlated to each other (Fig. 1D). However, *Novosphingobium* seemed to engage in a far more complex relationship with other genera than *Aeromonas*, with *Novosphingobium* having either a positive or negative relationship to 13 other genera, compared to *Aeromonas* being only positively or negatively associated with 4.

Taken together, these results indicated that in a natural water system with fluctuating nutrient supply, antibiotic exposure can significantly affect bacterial community succession. The persistence of *Novosphingobium* drew our attention in particular because of its resistance to CIP and its potential important role in the community. *Novosphingobium* is a common gram-negative bacterium that has been isolated from various environments, including lake waters, soils, plant tissues, and marine sediments (16). They are frequently found to be dominant in harsh and oligotrophic econiches and have been reported as common examples of CIP-resistant bacteria in aquatic environments (17). A high prevalence of antibiotic resistance has been reported for some *Novosphingobium* strains under CIP stress (18). Previous studies have identified *Novosphingobium* to be an ARG reservoir in various environments, including in drinking water, premise plumbing, and mangrove sediments (18–20), while it remains unclear what cellular and regulatory mechanisms enable these bacteria to survive under antibiotic stress.

### Involvement of iron homeostasis in the resistance of *Novosphingobium* to CIP stress

**Fig 2:**
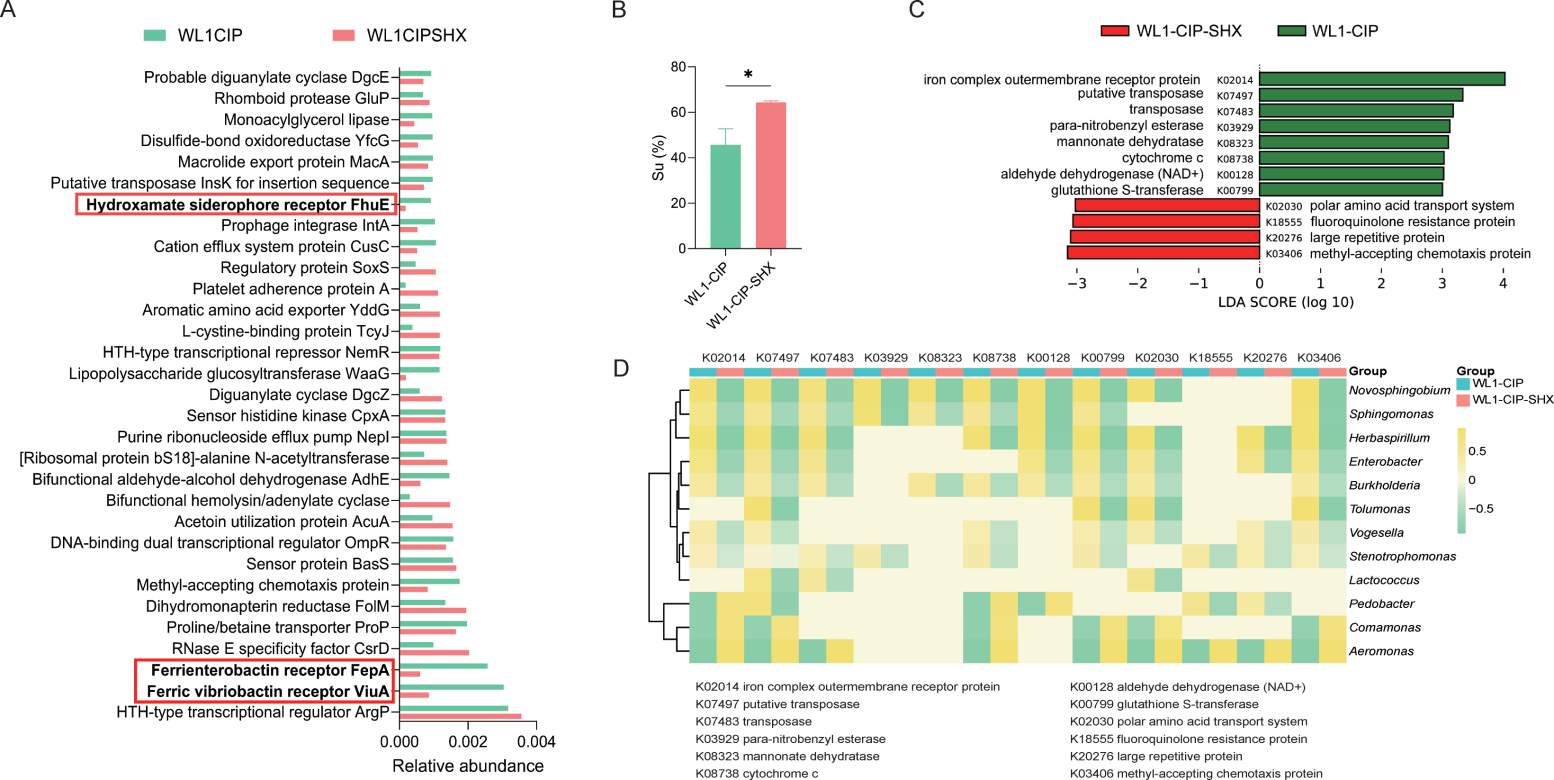
Meta-genomic analysis identified the involvement of iron homeostasis in CIP resistance of *Novosphingobium*. (**A**) Relative abundance of genes differentially enriched in different samples according to eggNOG classification. (**B**) Measurements of extracellular siderophore units of bacterial communities of different treatments. *P ≤ 0.05. (**C**) Histogram of the linear discriminant analysis (LDA) scores for differentially abundant functional genes analyzed by linear discriminant analysis effect size analysis (*P* ≤ 0.05, LDA  >  3). (**D**) Heatmap illustrating the abundances of genes in different KOs (KEGG Orthology) at the genus level.

The bacterial communities WL1-CIP and WL1-CIP-SHX were subjected to metagenome sequencing to evaluate the stringent response of the bacterial community under CIP stress. Metagenome sequencing results indicated that genes related to siderophore transport proteins such as FepA, ViuA, and FhuE were enriched in WL1-CIP (Fig. 2A). The proteins encoded by these genes are those involved in the initial step of iron uptake by binding siderophores and then facilitating the extraction of iron from the environment. The concentration of extracellular siderophores in WL1-CIP was significantly lower than that in WL1-CIP-SHX (Fig. 2B), suggesting that stringent response reduced the transportation of siderophores into bacteria under CIP stress.

The differential functional genes in the metagenomes of the two samples were further analyzed using the linear discriminant analysis effect size (LEfSe) method (21). LEfSe first provided the list of functional genes that were differential between WL1-CIP and WL1-CIP-SHX with statistical and biological significance and then ranked the 12 differential functional genes with the highest LDA values according to the effect size (Fig. 2C). It showed that genes related to iron complex outer-membrane receptor proteins and oxidative stress, such as cytochrome *c*, NAD^+^ oxidoreductase, and glutathione S-transferase, were significantly enriched in WL1-CIP. This result further supported the involvement of iron homeostasis during the stringent response. It also suggested that other metabolic activities such as cellular respiration and glutathione metabolism may be involved. To further identify the resources of these genes, taxonomic annotation was performed, and results indicated their highest abundances to be affiliated to *Novosphingobium* (Fig. 2D). This is consistent with the dominance of *Novosphingobium* in the sample WL1-CIP; however, the other dominant group, *Aeromonas*, showed a different trend. Notably, *Sphingomonas*, which belongs to the same family Sphingomonadaceae as *Novosphingobium*, demonstrated similar gene enrichment and clustered with *Novosphingobium*. Taken together, these data suggested the important roles of iron homeostasis in the persistence of *Novosphingobium* in the wetland microbiota, especially under sub-MIC CIP stress.

It has been well documented that the presence of antibiotics can alter the diversity, change the composition, and interfere with the function of bacterial communities in aquatic environments (22). However, responses of bacterial communities to antibiotics are also known to be highly heterogeneous due to the differences in antibiotics used and experimental conditions designed. To unravel such complexity, it is necessary to test the selection effect of individual antibiotics on certain taxa of bacteria under environmentally relevant conditions. In so doing, the mechanisms underlying the selection effect can then be elucidated.

The survival of bacteria in the presence of antibiotics may be ascribed to the acquisition of resistance, tolerance, and the formation of persistent cells (23). Bacteria gain resistance to antibiotic stress via various mechanisms including the activation of efflux pumps (24), the alternation in metabolic pathways, and the employment of stringent responses (25). The strategic mechanisms employed by the persistent bacterial cells are (p)ppGpp signaling (26), SOS response, toxin-antitoxin modules (27), and others (28). It had been reported that siderophores mediate iron homeostasis and endow antibiotic resistance in individual bacteria by regulating biofilm formation (29) or via inhibiting the oxidative stress response (30). The iron-dependent oxidative damage can even lead to cell death, a phenomenon initially described in eukaryotic organisms as ferroptosis (31). Although ferroptosis has never been rigorously defined in bacterial terms, ferroptosis-like death in prokaryotes has been proposed and accepted. Ferroptosis-like death in bacteria exhibits hallmark features of ferroptosis, including ferrous iron accumulation (32), ROS stress, GSH depletion (33), and MDA production due to lipid peroxidation (34). Both the stringent response and iron homeostasis are thereby known to exert regulatory effects on antibiotic resistance. Moreover, the stringent response has been reported to interact with iron homeostasis in regulating bacterial physiological activities, such as in prodiginine production (35). In this study, noting *Novosphingobium* to be resistant to CIP stress and to be persistent under stringent response conditions, we speculated that iron homeostasis may play a significant role in its survival. However, a clear connection and regulatory hierarchy among the stringent response, iron homeostasis, and CIP resistance in *Novosphingobium* has remained elusive. To answer this question, we employed *N. pentaromativorans* US6-1 (US6-1) for further investigation.

### Stringent response affects the growth and mortality of US6-1 under CIP stress

In the pilot experiments, the susceptibility of US6-1 and its derivatives to 17 kinds of antibiotics under both oligotrophic and eutrophic conditions was tested to construct an antibiotic resistance spectrum for US6-1 (Fig. S1). The MIC of CIP for US6-1 was measured as 16 µg/mL. In this work, the concentration of CIP added to the poor medium was set as 8 µg/mL to represent the sub-MIC concentration, mimicking the true environmental situation.

**Fig 3:**
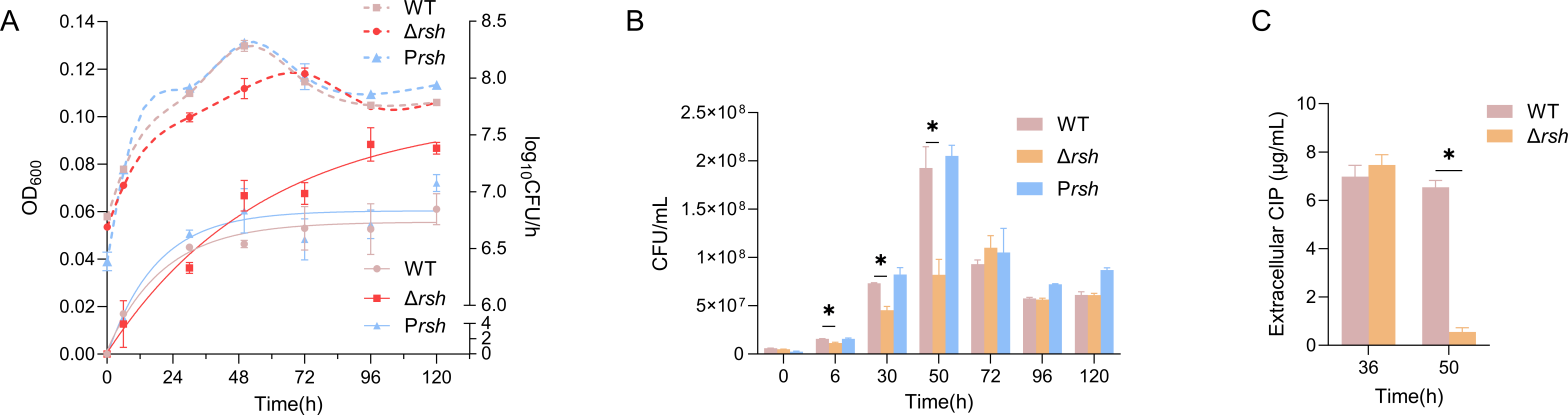
Stringent response affects the survival of *N. pentaromativorans* US6-1 under CIP stress. (**A**) Growth curves plotted by the biomass (left axis, solid line) and the viable and culturable population (right axis, dashed line) of US6-1 WT and its derivatives against cultivation time in oligotrophic medium with CIP treatment; (**B**) CFU counting of the WT and its derivatives in oligotrophic medium with CIP treatment; (**C**) measurements of extracellular CIP concentrations. Error bars represent the standard deviation. *P ≤ 0.05.

When exposed to 8 µg/mL CIP under oligotrophic conditions, the Δ*rsh* mutant exhibited a significantly different pattern in biomass accumulation when compared to the WT and P*rsh* strains. The latter two strains stopped biomass accumulation after 48 h, while the biomass of the Δ*rsh* kept increasing during the measurement period (Fig. 3A)‌. To assess bacterial survival under sub-MIC CIP stress, colony-forming unit (CFU) counts were quantified as a measurement for viable and culturable cells. ‌The growth curves were plotted by the log_10_ CFU/mL against time, and it was estimated that these strains entered the stationary phase at 50 h (Fig. 3A)‌. The Δ*rsh* mutant demonstrated significantly reduced CFU counts compared to the WT and P*rsh* strains at 30 h and 50 h, whereas no statistically significant difference was observed between the strains after 72 h (Fig. 3B), indicating that the WT and P*rsh* survived better than the Δ*rsh* mutant. This phenotypic divergence of biomass accumulation suggests that the absence of the stringent response mechanisms impairs the mutant’s ability to modulate its growth, possibly due to dysregulated metabolic adaptation to antibiotic-induced stress. The discrepancy between biomass and viable culturable cells suggested that the increased biomass in the Δ*rsh* mutant might be due to the accumulation of the dead cells or the formation of viable but nonculturable (VBNC) cells. It has been reported that stringent response contributes to the VBNC state as mutants lacking the capacity to synthesize (p)ppGpp exhibited a significantly reduced capacity to transition into the VBNC state (36). Consequently, the observed biomass accumulation in the Δ*rsh* mutant is more plausibly attributed to an elevated proportion of dead cells.

Measurement of extracellular CIP concentration indicated that there was no significant difference between the WT and the Δ*rsh* at 36 h, whereas at 50 h, the extracellular CIP concentration of the Δ*rsh* dramatically decreased to 0.55 µg/mL, indicating that most of the CIP in the culture medium had been transported into the Δ*rsh* cells during the exponential phase. (Fig. 3C).

These results demonstrated that the stringent response regulated the growth and mortality of US6-1 under CIP stress and mediated the transportation of CIP into cells. The stringent response was reported to promote rapid reallocation of cellular resources while slowing down the metabolic rate and increasing the functions required to facilitate survival (37). In US6-1 WT, the stringent response served as a brake at the exponential phase, slowing down the metabolic rate, increasing the survival rate, and decreasing the biomass of cells under CIP stress. This phenomenon was consistent with the significantly reduced abundance of *Novosphingobium* in WL1-CIP-SHX when compared to that of WL1-CIP. The decreased abundance of *Novosphingobium* in the bacterial community potentially allowed this genus to avoid antibiotic-induced death by entering a physiologically dormant state. On the other hand, the deletion of the stringent response gene *rsh* made it impossible for the Δ*rsh* mutant to slow down the metabolic rate in this way. Furthermore, the CIP was actively transported into cells, resulting in the significantly reduced survival rate of the Δ*rsh*. Once this had been confirmed, transcriptome sequencing was then performed to further investigate the biochemical mechanism underlying the regulation of the stringent response on cell viability and CIP transportation.

### Stringent response regulates global transcriptional activities, especially the core energy metabolism pathway

**Fig 4:**
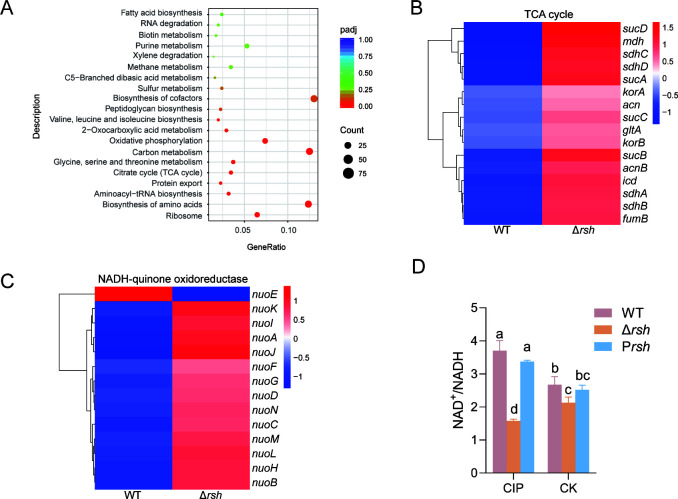
Transcriptome analysis reveals that stringent response regulates US6-1’s survival via the TCA cycle and oxidative phosphorylation under CIP stress. (**A**) Differentially expressed gene (DEG) statistics according to KEGG classification; (**B**) heatmap illustrating the expression levels of genes associated with the TCA cycle under sub-MIC CIP stress; (**C**) heatmap illustrating the expression levels of genes associated with the NADH-quinone oxidoreductase under sub-MIC CIP; (**D**) measurements of the relative NAD+/NADH ratio. Different letters above the bars indicate significant differences (*P* ≤ 0.05) among different treatments.

Comparative transcriptome analysis revealed that exposure to sub-MIC CIP induced substantial transcriptional reprogramming in the Δ*rsh* mutant, with 1,880 genes (approximately 37% of the US6-1 genome) exhibiting differential expression (|log_2_FC| > 1) relative to the wild-type strain. Among these, 920 genes were downregulated, while 960 genes were upregulated. Functional categorization of these differentially expressed genes (DEGs) highlighted their predominant enrichment in core energy metabolism pathways, including the ‌tricarboxylic acid (TCA) cycle‌ and ‌oxidative phosphorylation (Fig. 4A)‌.

The deletion of the stringent response gene *rsh* induced pronounced upregulation of core TCA cycle genes (Fig. S2) in US6-1 under sub-MIC CIP stress (Fig. 4B)‌. Specifically, *icd* (isocitrate dehydrogenase), *mdh* (malate dehydrogenase), *sucA* (2-oxoglutarate dehydrogenase E1), and *sucB* (2-oxoglutarate dehydrogenase E2) genes, encoding enzymes to catalyze the generation of NADH, all exhibited ~fourfold elevated expression in the Δ*rsh* mutant compared to the WT. Meanwhile, genes related to enzymes of the respiratory chain (Complex I–V) were upregulated under sub-MIC of CIP (Fig. 4C; Fig. S3). For example, 13 out of 14 core subunits of the respiratory chain Complex I (NADH dehydrogenase) were upregulated in Δ*rsh* (Fig. 4C).

To validate that the *rsh* deletion affected energy metabolism, NAD^+^/NADH ratios were quantified. The Δ*rsh* exhibited a 20% reduction of the NAD^+^/NADH ratio compared to the WT without CIP. Under CIP treatment, the Δ*rsh* mutant exhibited decreased NAD^+^/NADH ratios, whereas the WT strain showed increased ratios, underscoring the marked difference between the two (Fig. 4D). These results confirmed CIP’s interference with stringent response to regulate the energy metabolism in US6-1.

Overall, in US6-1 WT and derivatives, the activities of oxidative phosphorylation and the TCA cycle were all suppressed by stringent response under sub-MIC CIP stress to meet the balance of energy metabolism. The promotion of TCA cycle activity has been shown to result in increased cell respiration and drug uptake, which in turn leads to increased antibiotic susceptibility in *Pseudomonas aeruginosa* (38). It has also been reported that the resistance of *E. coli* to amoxicillin, enrofloxacin, and tetracycline was accompanied by a large set of DEGs related to cellular respiration and iron ion binding (39).

### Stringent response regulates iron homeostasis in US6-1 under CIP stress

**Fig 5:**
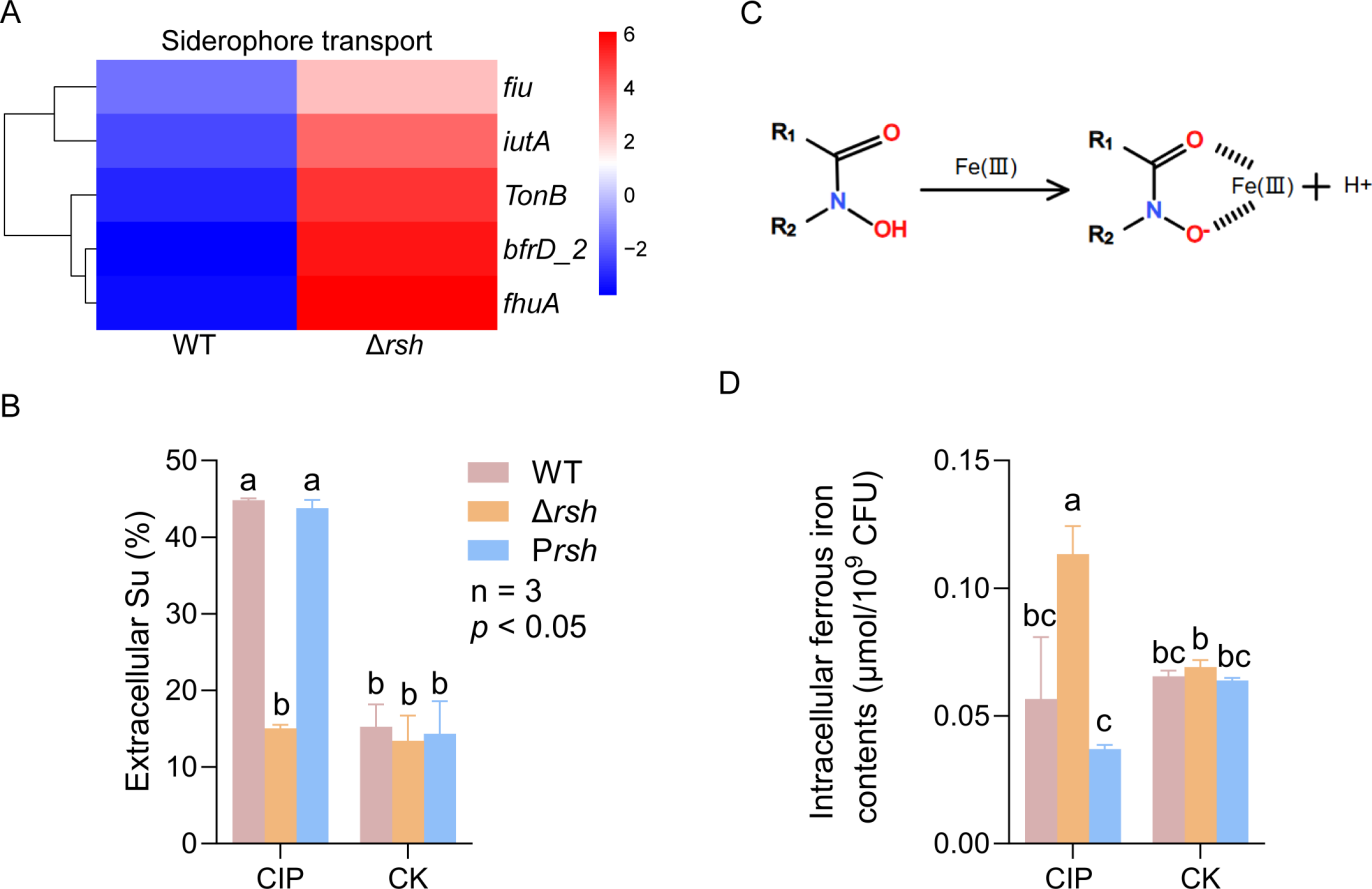
Stringent response affects iron homeostasis of US6-1 under CIP stress. (**A**) Heatmap illustrating the expression levels of genes associated with siderophore under sub-MIC CIP stress; (**B**) measurements of extracellular siderophore units; (**C**) the structure of hydroxamates and the complex of siderophore with iron; (**D**) measurements of intracellular ferrous iron contents. Different letters above the bars indicate significant differences (*P* ≤ 0.05) among different treatments.

Transcriptomic analysis identified significant upregulation of iron acquisition genes in the Δ*rsh* such as the siderophore receptor genes *fhuA*, *iutA, fiu*, and *bfrD*. Additionally, the expression of the *TonB* gene was also upregulated in Δ*rsh* (Fig. 5A). It is reported that the siderophores can bind with antibiotics, forming siderophore-drug conjugates; these siderophore-drug conjugates are recognized and bound by specific outer membrane receptors, including ferric hydroxamate uptake protein A (FhuA), ferric aerobactin receptor (IutA), and ferric iron uptake transporter (Fiu) (40–42); such complexes are subsequently transported into the bacterial periplasm via the TonB-dependent transport system, wherein TonB provides energy for transmembrane translocation through the proton motive force at the inner membrane (43).

Quantification of extracellular siderophore units during exponential growth revealed that without antibiotic treatment, there were no interstrain differences. However, upon CIP treatment, the extracellular siderophore units of the WT were significantly increased compared to the control cells, while the Δ*rsh* strain showed no significant changes (Fig. 5B). In observing the dramatic decline in the extracellular concentration of CIP in the Δ*rsh* strain, we assumed that the lower level of extracellular siderophore units in the Δ*rsh* mutant was due to the formation of siderophore-CIP conjugates under CIP stress. These findings align with reported mechanisms where enterobactin (Ent, a kind of siderophore) forms CIP-Ent conjugates to enhance antibiotic uptake‌ in *E. coli* (44). The siderophores can therefore act to enhance the cellular uptake of antibiotics by forming siderophore-antibiotic conjugates. Siderophores can be classified into hydroxamates, catecholates, and carboxylates (45). This “Trojan horse” antibiotic delivery strategy has been found in all three kinds of siderophores (46–48). However, direct biochemical validations of the siderophore-CIP conjugate, intracellular CIP concentration, and the TonB-dependent transport system in US6-1 remain to be done in the future.

FeCl_3_ experiments showed that siderophores produced by the US6-1 were hydroxamates (Fig. 5C). Previous studies have also revealed that *Novosphingobium* can produce siderophores to extract iron from the environment. However, the chemical property of the siderophores produced by *Novosphingobium* has not been identified (49, 50). This work revealed for the first time that the siderophores produced by *Novosphingobium* strain US6-1 were hydroxamates, a finding corresponding to the metagenome sequencing result (Fig. 2A) that showed genes related to hydroxamate siderophore receptor FhuE to be highly enriched in WL-CIP when *Novosphingobium* was the dominant group.

The concentrations of intracellular ferrous irons in the WT and its derivatives during the exponential phase were also measured. Under CIP stress, the deletion of the *rsh* resulted in a significant increment in intracellular ferrous iron content (Fig. 5D).

These results indicated that stringent response plays an important role in iron homeostasis of US6-1 under CIP stress. A stringent response might affect the iron uptake by reducing the formation and transportation of the siderophore-drug conjugates, resulting in decreased intercellular ferrous iron upon exposure to CIP. Ferrous iron has been confirmed as a major source of ROS production through the Fenton reaction (51). Perturbation of iron homeostasis promotes the evolution of antibiotic resistance in *E. coli* due to the elevated intracellular concentration of free iron and the consequent mutagenesis induced by oxidative damage (52). We propose that the significantly elevated intracellular ferrous iron concentration may promote ferroptosis-like death in the Δ*rsh* mutant.

### Stringent response mediates ferroptosis-like death in US6-1 under CIP stress

**Fig 6:**
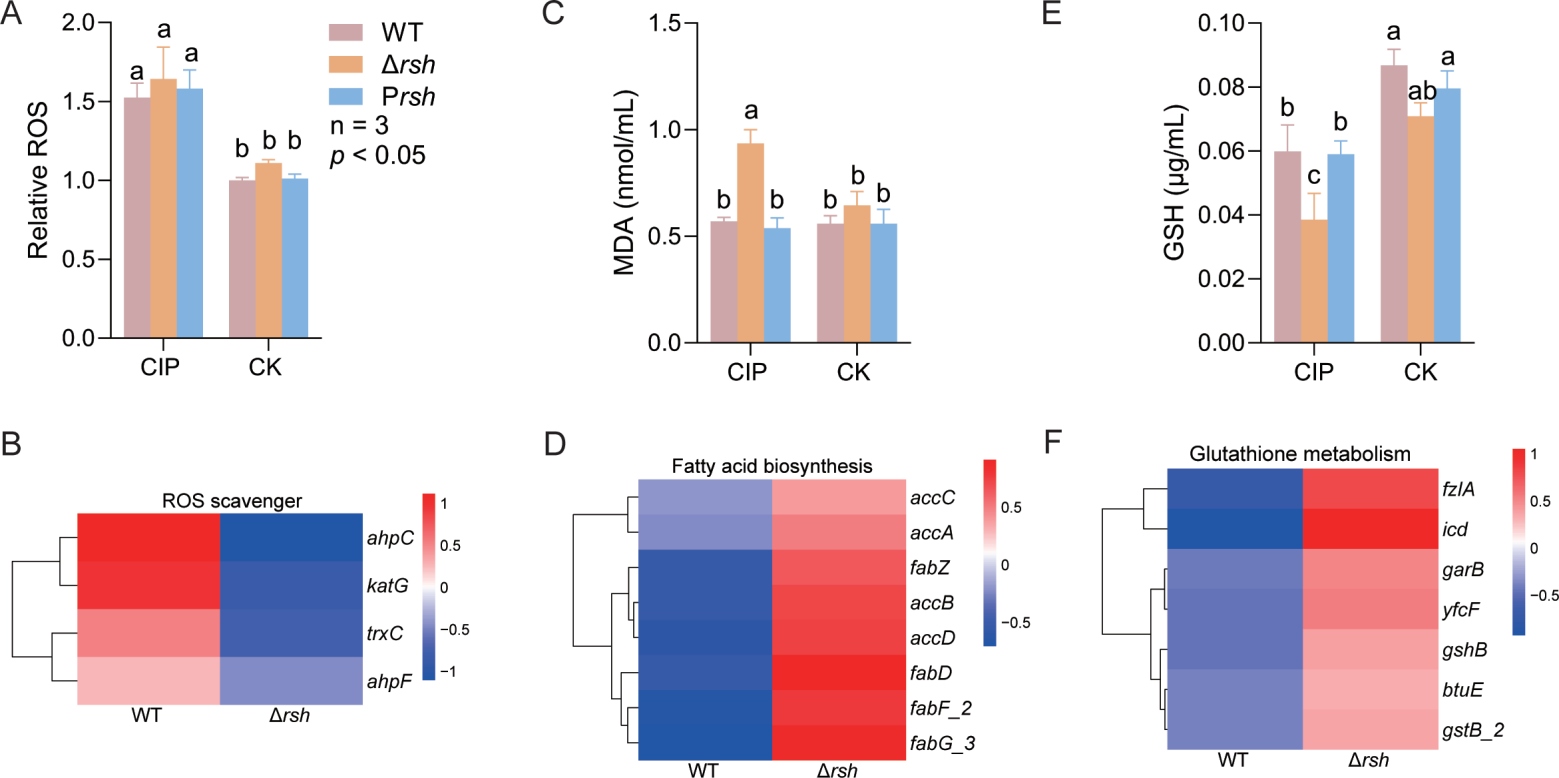
The stringent response may prevent US6-1 from ferroptosis-like death under CIP stress. (**A**) Measurements of reactive oxygen species (ROS); (**B**) heatmap illustrating the expression levels of genes associated with ROS scavengers under sub-MIC CIP stress; (**C**) measurements of malondialdehyde (MDA); (**D**) heatmap illustrating the expression levels of genes associated with fatty acid biosynthesis under sub-MIC CIP stress; (**E**) measurements of reduced glutathione (GSH); (**F**) heatmap illustrating the expression levels of genes associated with GSH metabolism under sub-MIC CIP stress. Different letters above the bars indicate significant differences (*P* ≤ 0.05) among different treatments.

Concentrations of ROS, MDA, and GSH were measured to evaluate whether the stringent response mediated ferroptosis-like death of US6-1 occurred under CIP treatments. Results indicated that the deletion of the gene *rsh* resulted in a slight but not significant increment of ROS, whereas CIP treatment significantly increased ROS in the WT and its derivatives (Fig. 6A). Expressions of the genes related to ROS scavengers such as alkyl hydroperoxide reductase AhpC and AhpF, catalase G, and KatG were all downregulated in the mutant (Fig. 6B), suggesting a lower capacity for the mutant when under antioxidative stress.

This was supported by the fact that, under CIP stress, the concentration of MDA in the mutant was significantly higher than that of the WT (Fig. 6C). MDA is one of the end products of lipid peroxidation and has been widely accepted as a biomarker for oxidative stress (53). Transcriptome analysis demonstrated that expressions of the genes related to fatty acid biosynthesis were all significantly upregulated in the mutant (Fig. 6D). We proposed that such biosynthesis of fatty acids had been stimulated in the Δ*rsh* mutant due to the interaction of ROS with fatty acids.

Coincidentally, CIP treatment significantly decreased GSH concentrations, particularly in the Δ*rsh* mutant (Fig. 6E). This suggests that the stringent response plays important roles in the buffering of antioxidant capacity. Transcriptome analysis indicated that the genes related to glutathione metabolism, such as glutathione synthetase GshB, glutathione peroxidase BtuE, glutathione reductase GarB*,* and glutathione S-transferases (GSTs), were all significantly upregulated in the Δ*rsh* strain (Fig. 6F). However, what their detailed contributions may be to glutathione homeostasis remains a question for further study.

**Fig 7:**
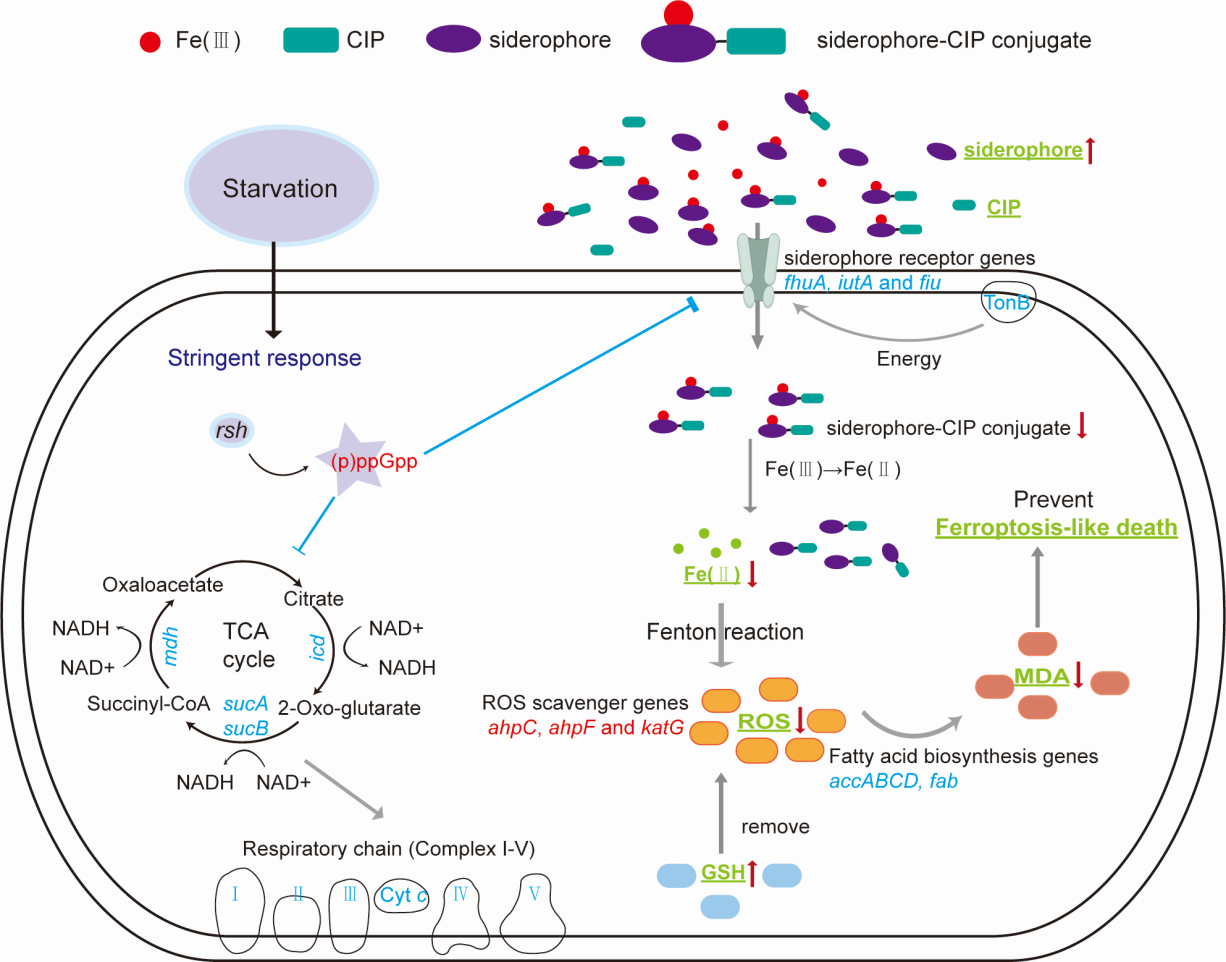
Schematic illustration of the role of stringent response in mediating ferroptosis-like death resistance through siderophore action under CIP stress. Genes in red color indicate upregulated expressions, and genes in blue color indicate downregulated expressions in US6-1 WT compared to that of the Δ*rsh*. The measured ferroptosis-like death hallmarks are highlighted in bold green with underlines. The red arrows indicate an upward or downward trend of the substances in the WT compared to that of the Δ*rsh*.

Figure 7 was constructed to provide an overall illustration of the stringent response-mediated ferroptosis resistance through siderophore action under CIP stress. In nutrient-limited environments, the gene *rsh* regulates the production of (p)ppGpp to induce the stringent response. The stringent response then inhibits various core pathways of cellular metabolism, including the TCA cycle and oxidative phosphorylation, to meet the balance of energy metabolism and slow down the growth rate. Additionally, the stringent response regulates iron homeostasis by significantly downregulating the TonB-dependent siderophore transportation, preventing the intracellular accumulation of Fe(II) and CIP, based on the assumption that the siderophores facilitate the entry of CIP and iron by forming conjugates (54). Furthermore, the stringent response upregulates the expressions of genes related to ROS scavenging, prevents the depletion of GSH, thus decreasing the MDA concentration resulting from lipid peroxidation. Therefore, we proposed that stringent response protects US6-1 WT from ferroptosis-like death under sub-MIC CIP stress. This study represents the first report of the stringent response mediating ferroptosis resistance through the siderophores under antibiotic stress.

The stringent response-mediated ferroptosis-resistance in US6-1 provides a good explanation for the persistence of *Novosphingobium* in the bacterial community under CIP stress. In the natural aquatic environment, the stringent response can inhibit core energy metabolism pathways to slow down growth rates. In addition, the stringent response mediates iron homeostasis and oxidative stress to reduce drug uptake and ferroptosis-like death, allowing *Novosphingobium* to enter a state of physiological dormancy to survive antibiotic exposure. The significance of *Novosphingobium* as potential carriers of ARGs had also been reported in microbial communities in river sediment (55) and drinking water distribution systems (56), highlighting the non-negligible ecological roles of *Novosphingobium*. Our work reveals possible mechanisms underlying the persistence of *Novosphingobium*, therefore establishing the stringent response or iron homeostasis as potential controlling targets for mitigating the propagation of antibiotic resistance in true oligotrophic environments.

## Data Availability

Gene sequences were submitted to NCBI under accession number PRJNA1198848. Raw transcriptome sequence data were submitted to NCBI under accession number PRJNA1164668.

## SUPPLEMENTAL MATERIAL

Supplemental material aem.01475-25-s0001.doc Figures S1 to S4; Tables S1 and S2.

ASM does not own the copyrights to Supplemental Material that may be linked to, or accessed through, an article. The authors have granted ASM a non-exclusive, world-wide license to publish the Supplemental Material files. Please contact the corresponding author directly for reuse.
