# Supplementary material for: Stringent response-mediated ferroptosis-like death resistance underlies Novosphingobium persistence during ciprofloxacin stress
Source: Appl Environ Microbiol. 2025 Sep 15;91(10):e01475-25. doi: 10.1128/aem.01475-25 (PMC12542694; doi:10.1128/aem.01475-25)
Supplement: Supplemental material — Figures S1 to S4; Tables S1 and S2. [file aem.01475-25-s0001.doc]

**Supplemental material**


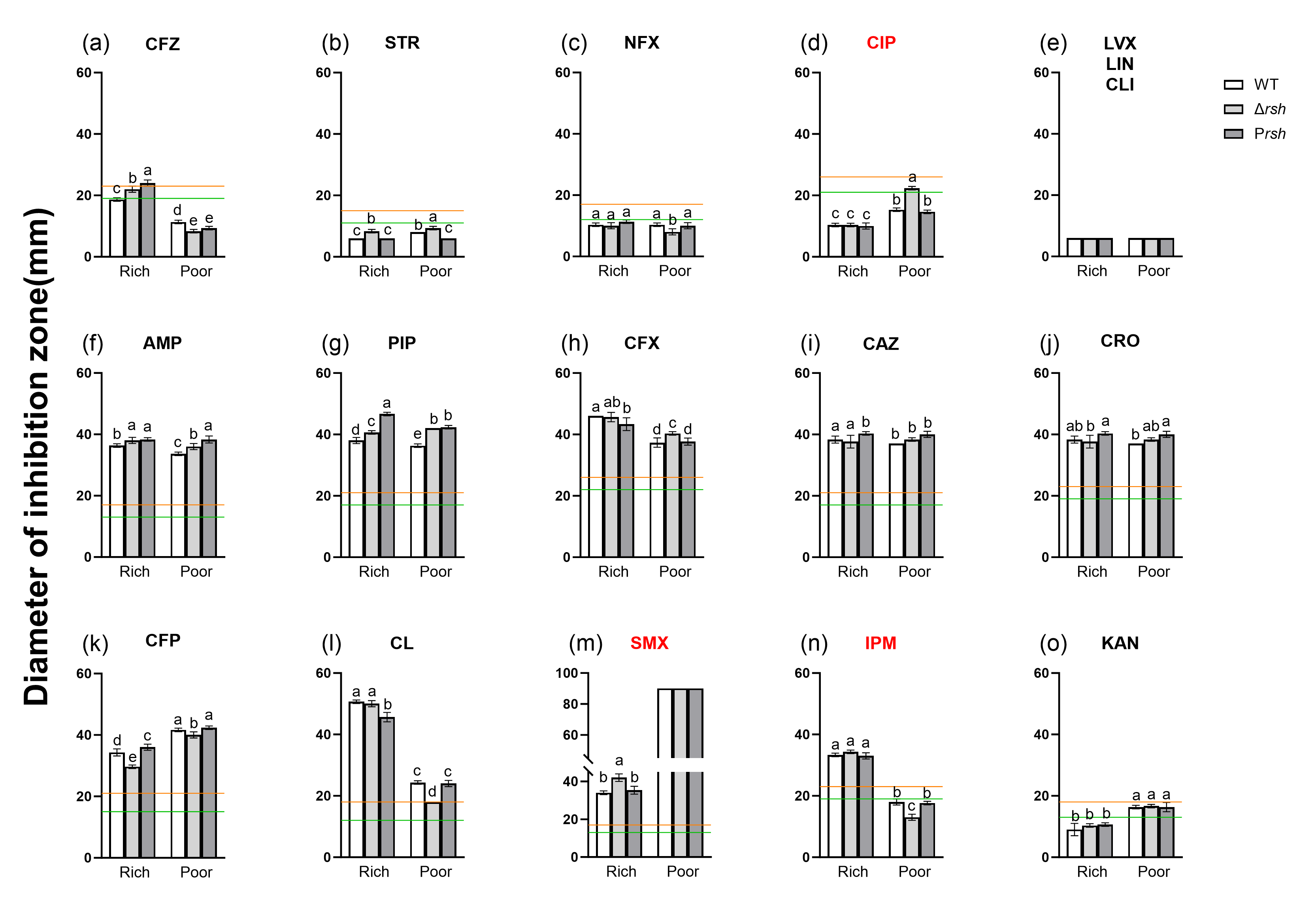


Figure S1. The diameter of the inhibition zone of different antibiotics against strains of US6-1. The green line represents “Resistant”； the orange line represents “Susceptible” according to CLSI. (A) CFZ: Cefazolin 30 µg; (B) STR: Streptomycin 10 µg; (C) NFX: Norfloxacin 10 µg; (D) CIP: Ciprofloxacin 5 µg; (E) LVX: Levofloxacin 5 µg; LIN: Lincomycin 2 µg; CLI: Clindamycin 2 µg; (the inhibition zones of LVX, LIN and CLI were not detected therefore all presented as the diameter of the antibiotic discs (6 mm) in the graph). (F) AMP: Ampicillin 10 µg; (G) PIP: Piperacillin 100 µg; (H) CFX: Cefuroxime Sodium 30 µg; (I) CAZ: Ceftazidime 30 µg; (J) CRO: Ceftriaxone 30 µg; (K) CFP: Cefoperazone 75 µg; (L) CL: Chloramphenicol 30 µg; (M) SMX: Sulfamethoxazole 250 µg; (N) IPM: Imipenem 10 µg; (O) KAN: Kanamycin 30 µg. Different letters above the bars indicate significant differences (P ≤ 0.05) among different treatments.


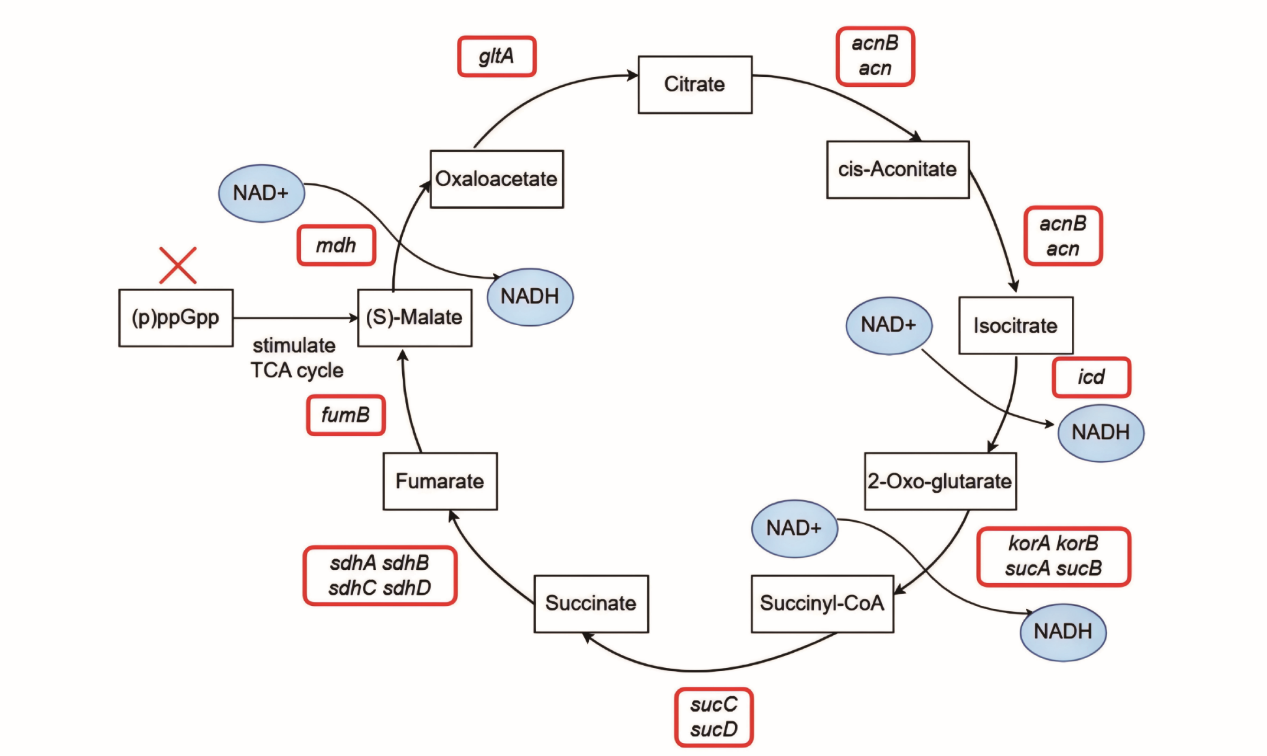


Figure S2. Schematic diagram of the TCA cycle in US6-1, red borders indicate genes involved in the TCA cycle, the red “X” indicates no (p)ppGpp in the mutant because of the deletion of the rsh gene, resulting in the stimulation of the TCA cycle.


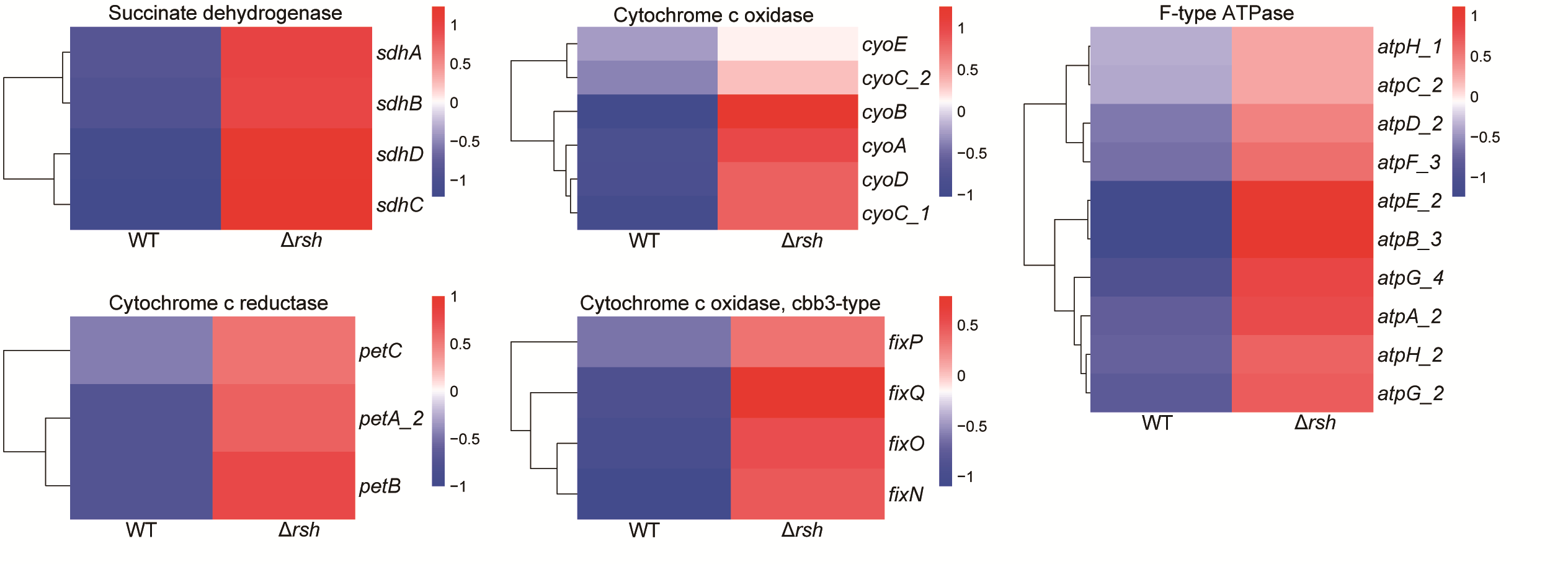


Figure S3. Heatmap illustrating the expression levels of genes associated with the enzymes on the respiratory chain (Complex Ⅱ-Ⅴ).

Table S1 Process of gradient elution

| Time/min | Flow phase A/% | Flow phase B/% |
| --- | --- | --- |
| 0.01 | 95 | 5 |
| 15.00 | 50 | 50 |
| 15.10 | 95 | 5 |
| 25.00 | 95 | 5 |

Table S2. List of Abbreviations

| Abbreviation | Definition |
| --- | --- |
| FLD | Ferroptosis-like death |
| CIP | ciprofloxacin |
| SR | stringent response |
| ARGs | antibiotic resistance genes |
| AMR | antimicrobial resistance |
| SHX | DL-Serine hydroxamate |
| ROS | reactive oxygen species |
| sub-MIC | sub-minimal inhibitory concentration |
| HPLC | high-performance liquid chromatography |
| LEfSe | Linear discriminant analysis Effect Size |
| ORF | open reading frame |
| AST | antimicrobial susceptibility testing |
| CLSI | Clinical and Laboratory Standards Institute |
| CFU | colony forming unit |
| VBNC | viable but non-culturable |
| ACN | acetonitrile |
| TFA | trifluoroacetic acid |
| DEGs | differentially expressed genes |
| CAS | Chrome Azurol S |
| GSH | reduced glutathione |
| NAD | nicotinamide adenine dinucleotide |
| MDA | malondialdehyde |
| HSD | Honestly Significant Difference |
| WT | wild-type |
| TCA cycle | ‌tricarboxylic acid cycle‌ |
| Ent | enterobactin |
| GSTs | glutathione S-transferases |
